# Macrophage Subsets and Death Are Responsible for Atherosclerotic Plaque Formation

**DOI:** 10.3389/fimmu.2022.843712

**Published:** 2022-03-30

**Authors:** Hongxia Li, Zhiqiang Cao, Lili Wang, Chang Liu, Hongkun Lin, Yuhan Tang, Ping Yao

**Affiliations:** ^1^ Department of Nutrition and Food Hygiene, School of Public Health, Tongji Medical College, Huazhong University of Science and Technology, Wuhan, China; ^2^ Hubei Key Laboratory of Food Nutrition and Safety, School of Public Health, Tongji Medical College, Huazhong University of Science and Technology, Wuhan, China; ^3^ Ministry of Education Key Laboratory of Environment, School of Public Health, Tongji Medical College, Huazhong University of Science and Technology, Wuhan, China

**Keywords:** atherosclerosis prevention, plaque formation, inflammation, macrophage polarization, macrophage death

## Abstract

Cardiovascular diseases, the notorious killer, are mainly caused by atherosclerosis (AS) characterized by lipids, cholesterol, and iron overload in plaques. Macrophages are effector cells and accumulate to the damaged and inflamed sites of arteries to internalize native and chemically modified lipoproteins to transform them into cholesterol-loaded foam cells. Foam cell formation is determined by the capacity of phagocytosis, migration, scavenging, and the features of phenotypes. Macrophages are diverse, and the subsets and functions are controlled by their surrounding microenvironment. Generally, macrophages are divided into classically activated (M1) and alternatively activated (M2). Recently, intraplaque macrophage phenotypes are recognized by the stimulation of CXCL4 (M4), oxidized phospholipids (Mox), hemoglobin/haptoglobin complexes [HA-mac/M(Hb)], and heme (Mhem). The pro-atherogenic or anti-atherosclerotic phenotypes of macrophages decide the progression of AS. Besides, apoptosis, necrosis, ferroptosis, autophagy and pyrotopsis determine plaque formation and cardiovascular vulnerability, which may be associated with macrophage polarization phenotypes. In this review, we first summarize the three most popular hypotheses for AS and find the common key factors for further discussion. Secondly, we discuss the factors affecting macrophage polarization and five types of macrophage death in AS progression, especially ferroptosis. A comprehensive understanding of the cellular and molecular mechanisms of plaque formation is conducive to disentangling the candidate targets of macrophage-targeting therapies for clinical intervention at various stages of AS.

**Graphical Abstract:**
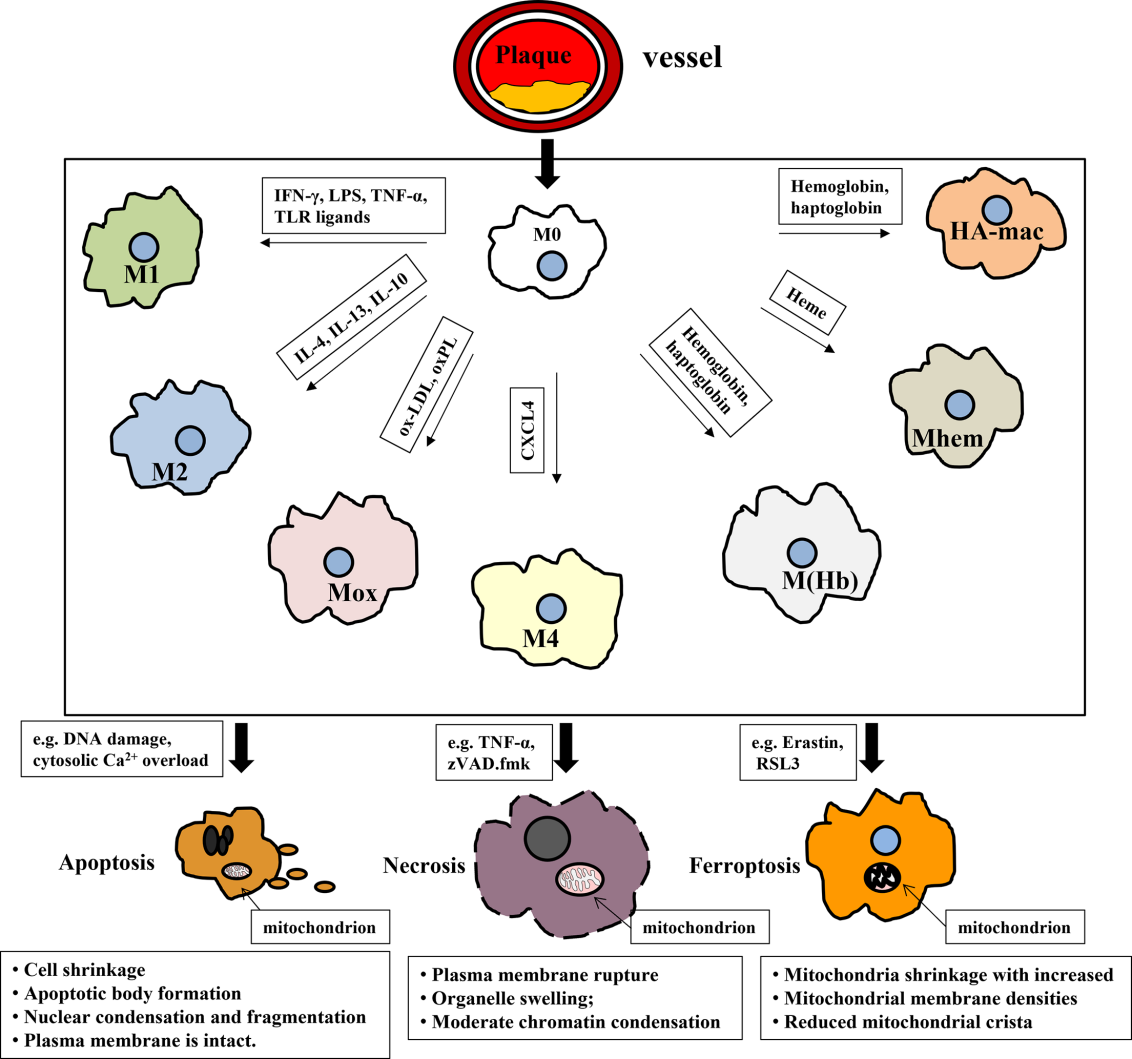
In atherosclerotic plaques, macrophages differentiate into different subtypes according to the vascular microenvironment. Subsequently, macrophages die from the inducement (iron or lipids overload, DNA damage) by the way of apoptosis, necrosis and ferroptosis etc., which, in turn, accelerates the atherosclerotic plaque formation.

## General Overview of Atherosclerosis

Cardiovascular disease (CVD) is the leading killer globally and has complex complications, such as stroke, myocardial infarction, heart failure, and hypertension (1). CVD has been a hot topic because of its high incidence and mortality. A report from the Global Burden of Disease Study showed that the total prevalence of CVD and years lived with a disability nearly have doubled from 271 million to 523 million and 17.7 million to 34.4 million in 1999–2019, and the cases of CVD deaths stably increased from 12.1 million to 18.6 million from 1990 to 2019 (2). What is more, the global trends for disability-adjusted life years and years of life lost also increased significantly (2–4). Atherosclerosis (AS), an underlying pathology of CVD, is a chronic, sterilized, inflammatory disease involved in large and medium arteries and accompanied by lipids, cholesterol, iron deposition, and fibrous cap thinning (5, 6). The occurrence and development of AS are strongly linked to local inflammation related to the vascular microenvironment and macrophage death.

Despite the fact that the exploration of cellular and molecular mechanisms has been inspiring over the last decades, the complex mechanisms of plaque formation in AS progression limit us in understanding the AS etiology comprehensively. Since the 19th century, researchers have thought of this disease related to the aging process (7). As the research further developed, three hypotheses have been formed. Firstly, it is the response-to-injury hypothesis (8). According to this hypothesis, various factors, including hemodynamic forces and pro-inflammatory factors, promote the dysfunction of the endothelium (8). Then, platelets aggregate, macrophages and vascular smooth muscle cells (VSMCs) engulf oxidized lipids and eventually form plaques, that is, the injury of vessel is mainly caused by endothelial detachment. However, subsequent studies have shown that the endothelium remains intact during the development of AS, even at the sites of plaque injury (9), which contradicts the core of this hypothesis. Secondly, it is the response-to-retention hypothesis. In the early stage of AS, proteoglycan binds to apolipoprotein B and traps lipids into macrophages (10). In addition, lipolytic enzyme and lysosomal enzyme play an important part in the residence of oxidized low-density lipoprotein (ox-LDL) in the vascular wall. *In vitro*, the lipoprotein lipase improved the adhesion of ox-LDL, leading to ox-LDL being more easily engulfed by macrophages and VSMCs and increasingly being foam cells (11). Thirdly, that is the oxidative modification hypothesis (12). Circulating LDL is chemically modified to ox-LDL by reactive oxygen species (ROS) and metal ions such as iron or copper, which becomes the pro-inflammatory factor in the vascular microenvironment. Macrophages and VSMCs internalize ox-LDL, an important atherogenic component of the vascular microenvironment *via* scavenger receptors, such as CD36, causing the transformation of foam cells containing cholesterol esters and subsequently to be the necrotic core.

As shown in **Table 1**, although the key factors for AS initiating are different among the three hypotheses, they are not mutually repulsive but stress diverse elements as the necessary and sufficient conditions to demonstrate the development of AS. For example, these hypotheses all consider that inflammation, a known feature of AS, is involved in atherosclerotic progression (16), and ox-LDL, an important component of the vascular microenvironment, is the key factor in promoting atherosclerotic lesions. Assuredly, the reduction of ox-LDL cholesterol is effective in AS treatment (7). Furthermore, these hypotheses all highlighted foam cell plays a critical part in every stage of AS, from the initial attack of the arterial lesion and lesion extension to cell death (apoptosis, necrosis, ferroptosis, etc.) causing plaque rupture and the complications of AS.

**Table 1:** Summary of three hypothesis in atherosclerosis progression.

Hypothesis	Inducers	Functioning molecules	Functioning cells	Similarities	References
Response-to-injury hypothesis	Platelets, oxidized lipids	PDGF, EDGF, PF4, CXCL4	Smooth muscle cells, endothelial cells, monocytes	Inflammation, ox-LDL, and foam cells promote atherosclerosis progression.	(8, 9, 12–15)
Response-to-retention hypothesis	Cholesterol, atherogenic lipoproteins	Sphingomyelinase, apoB-100, sulfotransferase, heparitinase	Macrophages, vascular smooth muscle cells		
Oxidative modification hypothesis	Oxidized lipids, like ox-LDL	RO2·, RO·, ·OH, ·NO, ·NO_2_, Fe, Cu, H_2_O_2_	Macrophages, vascular smooth muscle cells		

PDGF, Platelet-derived growth factor; EDGFs, endothelium-derived growth factors; CXCL4, CXC chemokine ligand 4; RO2**·**, RO**·**, peroxyl and alkoxyl radicals; PF4, platelet factor 4; **·**OH, Hydroxyl radical; NO, Nitric oxide; H_2_O_2_, Hydrogen peroxide; AS, atherosclerosis; NO_2_, nitrogen dioxide.

Recently, with the discovery of various polarization subsets of macrophages, the effect of different populations on the AS condition has been reexamined. Macrophage polarization and death, whose outcomes directly lead to plaque formation, are also considerable causes. Although there are inconsistent viewpoints on the mechanisms of occurrence and development of AS, the common denominator is the recognition of the essential role of inflammation, oxidized lipids, macrophage polarization, and macrophage death events in atherosclerotic vascular disease. This review focuses on inflammation and plaque formation in the initiation and development of AS and teases out the candidate proteins of macrophage-targeting therapies for clinical intervention at various stages of AS.

## Atherosclerotic Plaque Formation

The formation of atherosclerotic plaque is closely related to macrophages. Firstly, macrophages in plaques are differentiated from monocytes continuously recruited by chemokines, which is the prelude of macrophages transforming to foam cells. Subsequently, macrophages (M0) polarize into different macrophage subsets determined by the different vascular microenvironments. Macrophages with different phenotypes have different phagocytosic abilities to internalize ox-LDL; thus, the outcomes of being foam cells are distinct. Subsequently, foam cells undergo programmed or non-programmed death, causing the formation of plaques and necrotic cores, although different macrophage subsets may display unlike sensitivities for apoptosis, necrosis, ferroptosis, autophagy, and pyroptosis. Polarization and cell death play irreplaceable roles in plaque formation, and they also interplay with each other to control the AS condition. The two main inducements for plaque formation mentioned above are crucial for AS prevention and treatment and will be summarized below.

## Different Phenotypes, Different Outcomes

Macrophages display pro-inflammatory or anti-inflammatory properties due to different phenotypes. In the stimulation of cytokines, macrophages firstly migrate to the inflammatory sites to eliminate inflammation. However, in the process of AS, due to sustaining inflammation response, macrophages are continuously recruited to lesion sites. Monocytes firstly differentiate to macrophages (M0) due to cytokines, such as M-CSF (17). Although the differentiation of monocytes to macrophages is unreversible, macrophages possess eminent plasticity, i.e., switching their phenotypes and functioning according to external signals (18). As shown in **Table 2**, M0 could mainly polarize to seven types of macrophages according to the vascular microenvironment: classically activated macrophages (M1), alternatively activated macrophages (M2), oxidized phospholipid-induced macrophages (Mox), chemokine (C-X-C motif) ligand 4 or platelet factor 4-induced macrophages (M4), erythrocyte and hemoglobin-induced macrophages [HA-mac, M(Hb), and Mhem].

**Table 2:** Summary of the different macrophage subsets existing in atherosclerotic lesions of humans and mice.

Phenotype	Marker	Inducer	Products	Functioning molecules	Functions	Mouse/Human	References
M1	IL-1β, TNF-α, IL-6, IL-12, IL-23, CXCL9, CXCL10, CXCL11, Arg-2 (Mouse)	IFN-γ, LPS, TNF-α, TLR ligands, FFA, cholesterol crystals	iNOS, ROI, IL-12**↑**, IL-10**↓**, IL-23, IL-6, TNF-α, ROS	TLR4, IRAK4, TRAF6, IKK, NF-Kb, IRF3, STAT1, IRF5	Pro-inflammation, strong phagocytotic, strong migration	Human, Mouse	(19–24)
M2a	MR (Human), IL1Ra (Human), Arg-1(Mouse), FIZZ1 (Mouse), Ym1/2 (Mouse)	IL-4, IL-13	IL-10, TGF-β, CCL22, CCL17	TLR2, STAT6, Trim24, NEAT1, miR-224-5p	Anti-inflammatory, tissue remodeling, endocytosis	Human, Mouse	(19, 25–27)
M2b	IL-10**↑**, IL-12**↓**	IC+LPS/IL-1β	IL-10**↑**, IL-12**↓**, TNF-α, IL-6	STAT6, Trim24	Immunoregulation	Human, Mouse	(19, 25)
M2c	MR (Human), Arg-1(Mouse)	IL-10, TGFβ, glucocorticoids	IL-10, TGF-β, PTX3	STAT6, Trim24	merTK-dependent efferocytosis	Human, Mouse	(19, 25)
M2d	TNF-α**↓**, IL-12**↓**	TLR+A_2_R agonists	VEGF, IL-10, iNOS	STAT6, Trim24	Pro-angiogenic, tumor promotion	Mouse	(19, 25)
Mox	HO-1, Srxn1, TrxR1, Nrf2	ox-LDL/oxidized phospholipids	IL-10, IL-1β, HO-1	Nrf2, HO-1, TrxR1	weakly phagocytotic, weakly migration	Mouse	(26, 28, 29)
M4	MMP7, S100A8, MR	CXCL4	MMP12, IL-6, TNF-α	CXCL4, CD163, HO-1	Weakly or no phagocytic, almost no foam cell formation	Human	(26, 29, 30)
M(Hb)	CD163, MR, LXRα	Hemoglobin/haptoglobin	ABCA1, ABCG1, LXRα	LXRα, CD163, ferroportin	Hemoglobin phagocytoses, strongly cholesterol efflux	Human	(26, 29, 31, 32)
Mhem	CD163, ATF1	Heme	LXRβ	CD163, LXRβ, HO-1, ATF1, AMPK	Anti-atherogenic, erythrophagocytosis	Human, Mouse	(30, 31, 33)
HA-mac	CD163**↑**, HLA-DR**↓**	Hemoglobin/haptoglobin	HO-1	CD163, IL-10	Anti-atherogenic, hemoglobin clearance	Human	(30, 31)

↑, high; ↓, low; CXCL, C-X-C motif chemokine; FFA, free fatty acid; FIZZ1, found in inflammatory zone 1; Ym1, T lymphocyte-derived eosinophil chemotactic factor; HO-1, heme oxygenase-1; PTX3, pentraxin-3; IKK, inhibitor of kappa B kinase; ROI, reactive oxygen intermediates; IRF3, interferon-responsive factor 3; STAT6, signal transducer and activator of transcription 6; IFN-γ, interferon-γ; IL, interleukin; TLR, Toll-like receptor; TGF, transforming growth factor; MR, mannose receptor; A_2_R, adenosine receptor A2; LXR, liver X receptor; VEGF, vascular endothelial growth factor; MerTK, Mer receptor kinase; CCL, chemokine (C-C motif) ligand; MMP, matrix metalloproteinase; S100A8, S100 calcium-binding protein A8; ATF1, cyclic AMP-dependent transcription factor 1; Srxn1, sulfiredoxin 1; Txnrd1, thioredoxin reductase 1; TNF, tumor necrosis factor; ABCA1, ATP-binding cassette transporter A1; HLA-DR, human leukocyte antigen DR.

As illustrated in **Table 2**, M1 macrophages, highly expressed iNOS, are differentiated from the M0 phenotype by lipopolysaccharide (LPS), interferons, pathogen-associated molecular patterns, and lipoproteins *via* Toll-like receptor signaling, especially TLR4/MyD88/NF-κB (34). Moreover, TIR domain-containing adaptor inducing interferon-beta (TRIF), another downstream protein of TLR4, is also involved in M1 activation except for MyD88. TRIF could activate the transcription factor interferon-responsive factor 3 (IRF3) and then promotes IFNα and IFNβ secretion. Therefore, interferons bind to the interferon receptor (IFNAR) to activate the transcription factor STAT1 to skew macrophages to M1-like or M1 polarization (35) M1 macrophages are pro-inflammatory to destruct tissue and secrete cytokines, for example, IL-1β, IL-6, TNF-α, and IL-12 (36). Pro-inflammatory cytokines sustain to recruit immune cells, causing many macrophages to migrate to lesion sites. Compared with M0, the phagocytosis capacity of M1 is not inferior; even M1 macrophages are more likely to be foam cells, an indispensable part of plaques. If foam cells die and could not be removed, they will become new pro-inflammatory factors, which becomes a vicious circle, that is, recruitment–death–recruitment.

M2 macrophages with high expression of arginase-1 could be polarized by the cytokines like IL-4 and IL-13, and subsequently divided into M2a, M2b, M2c, and M2d, depending on the stimulation (37). As shown in **Table 2**, IL-4 or IL-13 induces macrophages to the M2 subset through activating STAT6 by the IL-4 receptor α, and IL-10 induces macrophages to M2 polarization *via* STAT3 through the IL-10 receptor (35). Recently, Cao and colleagues reported that the knockdown of the long noncoding lncRNA-MM2P reduced cytokine-driven M2 polarization and M2-marker genes by decreasing phosphorylation on STAT6 (38). M2 macrophages initiate anti-inflammatory signaling by generating cytokines like the IL-1 receptor agonist, collagen, IL-10, and TGF-β1, making M1 transform into M2 macrophages to strengthen exocytosis (39). M2 macrophages are differentiated by Th2 cytokines and generate IL-10. Due to various scavenger receptors such as CD36, macrophage scavenger receptor 1 (MSR1), macrophage receptor with collagenous structure (MRC1), and mannose receptor (40, 41), the phagocytosis of M2 macrophages is rather strong, causing the phagocytosis capacity of M2 to be superior to M0 (28). It means that if M2 could not well cope with intracellular lipids, they will be a hidden danger for plaque formation due to their “greed.”

Mox is polarized from M0 by oxidized phospholipids and keeps them away from oxidative stress *via* nuclear factor E2-related factor 2 (Nrf2)-regulated expression of antioxidant enzymes like HO-1, thioredoxin reductase 1 (TrxR1), and sulfiredoxin-1 (Srxn1) (28). In atherosclerotic lesions of ldlr^-/-^ mice, Mox macrophages were extensively distributed in plaque and account for 30% while M1 and M2 macrophages took up 40% and 20% of all plaque macrophages, respectively (28). Notably, the phagocytosis and migration capacities of Mox are inferior to M1 and M2 subsets. What is more, antioxidant proteins were dramatically upregulated by Nrf2 in Mox, suggesting that Mox macrophages may be an anti-atherosclerotic subset. However, it is unclear whether Mox is pro-atherosclerotic or anti-atherosclerotic in AS progression until now. Certainly, M1, M2, and Mox macrophages are all present in atherosclerotic lesions, and the imbalance of the ratio of macrophage subsets may be the cause of plaque formation and the impediment of inflammation alleviation (42).

Iron-loaded M4 macrophages are induced by CXC chemokine ligand 4 (CXCL4) (43). The M4 phenotype is found in human plaque lesions and marked by matrix metalloprotease 7 (MMP-7) and Ca^2+^-binding protein S100A8 (44). The combination of CD68, S100A8, and MMP7 is a reliable marker to recognize the M4 subset both *in vitro* and *in vivo (* 43). The M4 subset is dominatingly presented in the adventitia and intima of human arteries to trigger inflammation and promote plaque instability. Moreover, CD163 alleviates AS progression by upregulating the atheroprotective enzyme HO-1 in response to hemoglobin. It was reported that CXCL4 aggravated AS by suppressing CD163 and scavenger receptors CD36 or SR-1, which was consistent with the conclusion in ApeE^-/-^ mice (45, 46), indicating the proatherogenic effects of this population.

As illustrated in **Table 2**, HA-mac, M(Hb), and Mhem populations display in the hemorrhagic sites of unstable plaques where they engulf and recycle erythrocyte remnants and hemoglobin, and could be induced by hemoglobin, haptoglobin, and CD163. All of them are regulated by CD163, but HA-mac, M(Hb), and Mhem are also activated by IL-10, LXRα, and ATF1/MAPK, respectively (32). Because of the high expression of LXRa, LXRβ, ABCA1, and ABCG1 which are responsible for the cholesterol efflux, HA-mac, M(Hb), and Mhem subsets are atheroprotective and resist to be foam cells (32, 47), but their effects on AS protection are limited because of the small percentage in plaque lesion.

## Macrophage Death, Efferocytosis Impairment, the Culprit of Plaque Formation

Cell death and dead cells not being removed effectively are other major causes of plaque and necrotic core formation. The programmed death of macrophages is a complex process involving multiple mechanisms, such as endoplasmic reticulum stress (48), oxidative stress, mitochondrial dysfunction, and lysosome rupture (49). As displayed in **Table 3**, atherosclerotic plaques contain a large amount of cholesterol as well as necrosis cores comprised of foam cells, collagen, smooth muscle cells, etc. (99), due to the dysfunction of the death-clearance mechanism on macrophage apoptosis, necrosis, and ferroptosis (100, 101). For macrophage death and clearance, the effects of apoptosis–efferocytosis, necrosis, ferroptosis, autophagy, and pyroptosis are highlighted below.

**Table 3:** Cell death types contributed to plaques and necrosis core in atherosclerosis.

Death types	Defining morphological features	Functioning molecules	Inducers	Inhibitors	References
Apoptosis	Plasma membrane blebbing; cellular and nuclear volume reduction; nuclear fragmentation	Caspase-1, CARD8, GZMB; HSP70, CARD6, NOX5, PI3K/Akp53, Bax, Bak, Bcl-2, Bcl-XL	UNC5B, multiple intracellular stress conditions (e.g., DNA damage, cytosolic Ca^2+^ overload), apoptozole, FASL, DCC, perillyl alcohol	XIAP, ML-IAP/livin, NAIP, ILP-2, Bruce/Apollon, c-IAP1, surviving, c-IAP2, Z-VDVAD-FMK	(50–59)
Necrosis	Plasma membrane rupture, organelle swelling, moderate chromatin condensation	MPG, CA9, RIP1, MLKL, PDE4, RIP3, DCC1, CD40, MLKL, COL4A3BP	TNF-α, PF-543, TNF-α-IN-2, PF-543 Citrate, fasentin	Necrostatin1 (Nec-1), IM-54, necrosulfonamide (NSA), myristoleic acid	(60–69)
Ferroptosis	Mitochondria shrinkage with increased mitochondrial membrane densities, reduced mitochondrial crista	GPx4, FSP1, DHODH, RPL8, IREB2, ATP5G3, ACSF2, P53, HSPB1, SLC7A11, VDACs, Nrf2, xCT	RSL3, DPI7, erastin, DPI10, DPI13, DPI12, DPI18, ML16, DPI17, sorafenib, DPI19, artemisinin derivatives	Desferoxamine, solamine, 2, 2-bipyridyl vitamin E, U0126, trolo, ferrostatin-1, SRS8-24, SRS8-72, SRS11-92, SRS12-45, SRS13-35, SRS13-37, SRS16-86, CA-1	(69–78)
Autophagy	Extensive cytoplasmic vacuolization, autophagosome formation, phagocytosis, lysosomal degradation	LC3-I, LC-II, Atg-5, Atg-7, Atg-9, Beclin1, P62, SQSTM1, Rb7, TFEB, SR-BI, ABCA1, PPARalpha, AMPK	Ox-LDL, 7-hydroxy cholesterol, free cholesterol, cholesterol crystals, ROS, tomatidine, metformin, trimetazidine, crustecdysone, syringin	Proteases E64, concanamycin A, typhaneoside, liensinine diperchlorate, liensinine, cycloheximide	(79–90)
Pyroptosis	Necrosis-like cell-membrane pore formation, cellular swelling, membrane rupture, massive leakage of the cytosolic contents, apoptosis-like nuclear condensation, DNA fragmentation without DNA laddering	GSDMD, NLRP3, IL-1β, HMGB1, ASC, TLR4, NF-κB, caspase-1, caspase-11, caspase-3, caspase-8	Double-stranded DNA, LPS, ox-LDL, uric acid crystals, extracellular ATP, cholesterol crystals, ROS, nicotine, acrolein, TNF-α, triglyceride, salmonella	Disulfiram, quercetin, succination	(91–98)

ROS, reactive oxygen species; RSL, Ras-selective lethal 3 compound; SAS, sulfasalazine; VDACs, voltage-dependent anion channels; Bcl-2, B-cell lymphoma-2; CARD8, caspase recruitment domain-containing Protein 8; Bcl-XL, B-cell leukemia/lymphoma XL; GZMB, Granzyme B; MLKL, mixed lineage kinase domain-like pseudokinase; HSP70, heat shock protein; Bak, Bcl-2 homologous antagonist/killer; CARD6, caspase recruitment domain-containing protein 6; NOX5, nadph oxidase 5; Bax, Bcl-2-associated X protein; RIP1, receptor-interacting protein 1; FASL, Fas ligand; DCC, Deleted in colon cancer; UNC5B, Unc-5 netrin receptor B; COL4A3BP, collagen type IV alpha 3 binding protein; RIP3, receptor-interacting protein kinase 3; EDD1, embryo defective development 1; RPL8, ribosomal protein L8; MPG, N-(2-mercaptopropionyl) glycine; CA9, carbonic anhydrase IX; SIRT5, sirtuin 5; DCC1, DNA replication and sister chromatid cohesion 1; ACSF2, acyl-CoA synthetase family member 2; VDACs, voltage-dependent anion channels; IREB2, iron responsive element binding protein 2; CS, citrate synthase; ATP5G3, ATP synthase subunit 9, isoform 3; HSPB1, heat shock protein family B member 1; SLC7A11, cystine/glutamate antiporter solute carrier family 7 member 11; VDACs, voltage-dependent anion channels; GSDMD, gasdermin D; HMGB1, high-mobility group box-1; ASC, apoptosis-associated speck-like protein.

## Macrophage Apoptosis and Efferocytosis in Atherosclerotic Plaque Formation

Cell apoptosis has been regarded as a pivotal step for necrotic core formation and unstable plaque rupture. High levels of ox-LDL and cholesterol overload-induced ERs cause macrophage apoptosis (102). Apoptosis executes the programmed progression, which is the activation of caspase-type proteases (103). Macrophage apoptosis is the main cause of necrosis cores and adverse remodeling of the plaque architecture, leading to vulnerable plaques (104). What is more, epidemiological studies showed that AS could be aggravated by hyperhomocysteinemia, which induced inflammation, lipid accumulation, and macrophage apoptosis in arteries (105). However, some cytokines, like colony-stimulating factor 1 (CSF1) derived from VSMCs and endothelial cells promoted macrophage proliferation and reduced macrophage apoptosis in plaques (106), suggesting that vulnerable plaque formation may be effectively prevented by inhibiting macrophage apoptosis. On other hand, because the phagocytic receptors fall off the macrophage cell membrane, in comparison with other human macrophage-rich tissues, efferocytosis is impaired in atherosclerotic plaques (107). This impairment not only decreases the phagocytic capacity but also generates molecules to compete with macrophages for the identification of apoptotic cells, which reduces the clearance of apoptotic cells and subsequently intensifies the inflammatory response (107, 108). Macrophage apoptosis is exacerbating, but efferocytosis is impaired, which enhances atherosclerotic plaque formation under the stimulation of pro-inflammatory factors.

## Macrophage Necrosis in Atherosclerotic Plaque Formation

Apoptosis has been concerned with atherosclerotic plaque formation for several decades. This programmed cell death causes the clearance of unhealthy cells but does not produce detrimental substances to the microenvironment. The outcomes of apoptosis closely rely on the stage of the AS (109). It is worth noting that apoptotic macrophages on the early stage of plaques are efficiently cleared by efferocytosis; thereby, secondary necrosis could be prevented. However, efferocytosis is tremendously defective in advanced atherosclerotic plaques; thus, apoptotic macrophages aggregate and secondary necrosis occurs (110). Ox-LDL stimulation, mitochondrial ROS overproduction, ATP depletion, intracellular Ca^2+^ overload as well as impaired efferocytosis are all inducements to trigger macrophage necrosis. Macrophage necrosis leads to plaque vulnerability and releases massive pro-inflammatory cytokines and DAMPs (damage-associated molecular patterns) like high-mobility group box 1 (HMGB1), heat shock proteins as well as S100 family molecules (109). The result of transmission electron microscopy showed that VSMCs (30 ± 18%) were observed to occur necrosis but not apoptosis (1 ± 2%) in advanced human plaques. Moreover, dying macrophages present the necrotic cell morphology with membrane disruption and swollen, disintegrating organelles (110), implying that macrophage necrosis plays a considerable part in advanced plaques. Recently, GPR32, a receptor for pro-resolving lipid mediators like resolvin D1, was reported to be reduced in human atherosclerotic lesions. Exhilaratingly, the lesion area and necrosis of atherosclerotic plaques were observably decreased when this receptor was overexpressed in mice (111). However, because the applied detection methods for necrosis have not been found, it has not been investigated extensively as apoptosis.

## Ferroptosis, “an Up-Rising Star” in Atherosclerotic Plaque Formation

The term ferroptosis was defined to explain the manner of cell death stimulated by the chemical reagent erastin (112). Once the concept of ferroptosis was put forward, researchers paid great attention to it in the field of cancer treatment. Until now, the study of ferroptosis is still limited in AS, especially in macrophages. The differences of ferroptosis in cancer and AS are as follows: firstly, the pivotal challenge is to efficaciously kill cancer cells and keep the healthy cells impervious in cancer research, while macrophage ferroptosis would like to be prevented in AS (113). Secondly, cancer cells are defective in cell death executioner mechanisms including ferroptosis resistance but macrophages are more susceptible to ferroptosis. To enable growth, cancer cells exhibit an increased nutrient demand including iron and lipids compared with macrophages (113); therefore, cancer cells could be more capable of solving crises caused by iron and lipid overload to escape ferroptosis (114). However, due to plaque rupture, iron and lipids are overloaded in AS; macrophages are suffering the challenge from both lipids and iron-containing proteins such as hemoglobin, which could induce macrophage ferroptosis (115).

**Figure 1:**
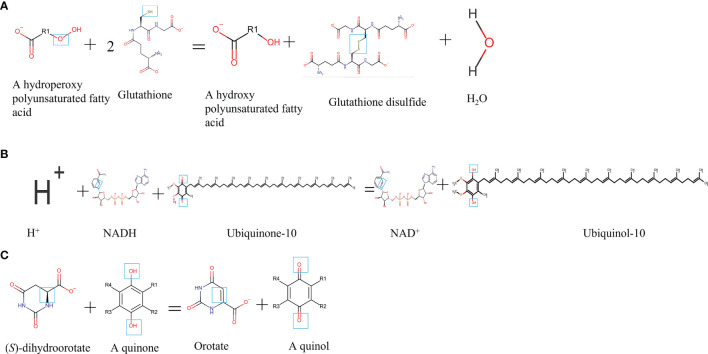
Chemical formula and catalytic activity GPx4, FSP1, and DHODH in ferroptosis regulation. **(A)** GPx4. **(B)** FSP1. **(C)** DHODH. NADH, reduced form of nicotinamide-adenine dinucleotide; NAD+, nicotinamide adenine dinucleotide.

**Figure 2:**
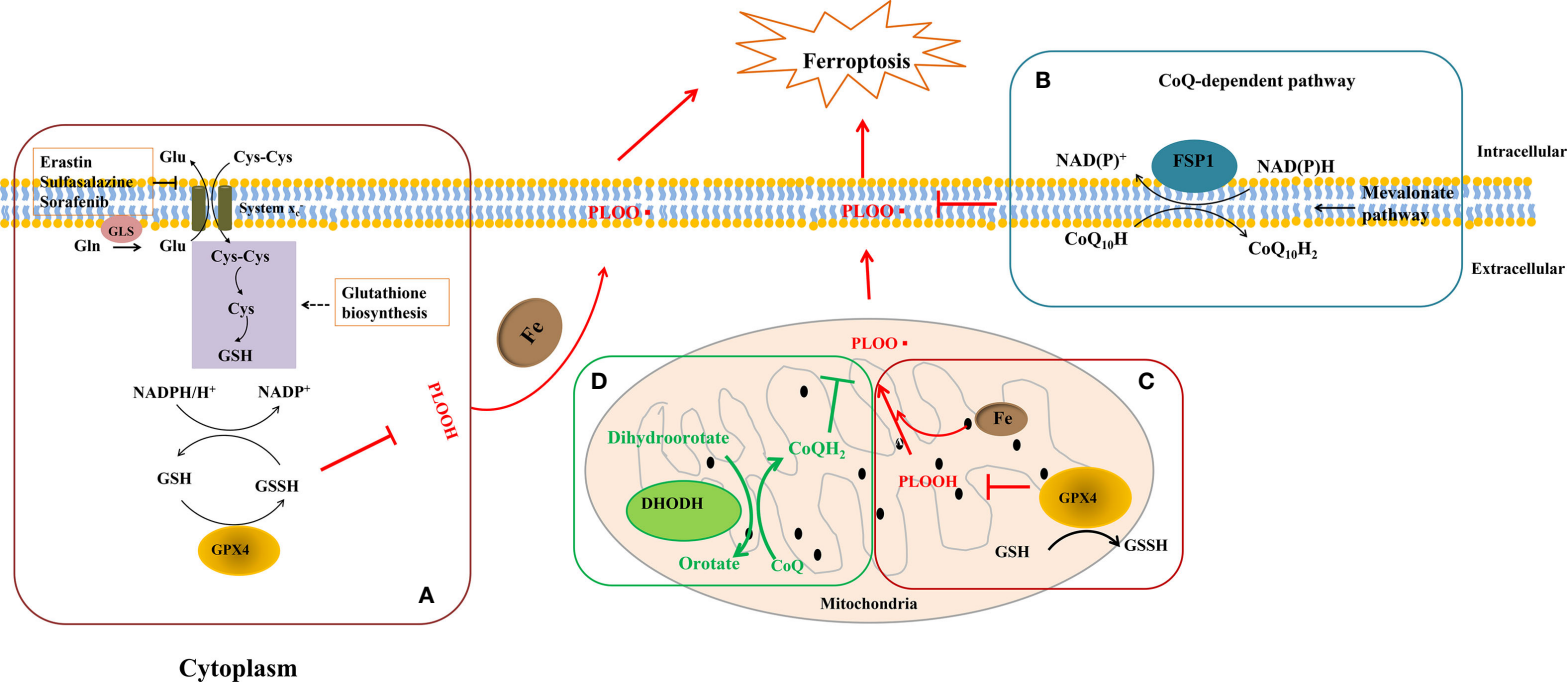
GPx4, FSP1, and DHODH on cell ferroptosis. **(A)** Firstly, cystine (Cys-Cys) was transported for keeping the homeostasis. Meanwhile, L-glutamine (Gln) was catalyzed by glutaminase (Gls) to become L-glutamate (Glu) and outputted through system Xc- as well. Secondly, GPx4 oxidizes GSH to GSSG to reduce lipid peroxidation in cytoplasm and mitochondria. **(B)** FSP1 reduces ubiquinol-10 by catalyzing coenzyme Q/ubiquinone-10, which is a process independent of the GPX4 at the plasma membrane. **(C, D)** In the mitochondrial inner membrane, DHODH operates in parallel to mitochondrial GPx4 to inhibit ferroptosis and catalyzes the conversion of dihydroorotate to orotate *via* catalyzing the reduction of coenzyme Q/quinone to quinol. NAD(P)H, nicotinamide adenine dinucleotide phosphate; NAD+, nicotinamide adenine dinucleotide; PLOOH, phospholipid hydroperoxide.

Ferroptosis, showing the iron-dependent excessive generation of lipid ROS accompanied with the depletion of plasma membrane polyunsaturated fatty acids, has been found to promote the formation and destabilization of plaques (116, 117). Ferroptosis not only controls the death but also the phenotype of macrophages. Macrophages in the vascular microenvironment with excess iron and ox-LDL or LPS/IFN-γ, the proportion of M1 and Mox macrophages increases (115). What is noteworthy is that, compared to M2 macrophages, M1 and Mox macrophages display the ability of ferroptosis resistance (115), implying the interplay between macrophage ferroptosis and polarization. Because lipid peroxidation and iron overload are common and obvious characteristics of plaques, ferroptosis does play a non-negligible role in AS progression (117, 118). Recently, the glutathione (GSH)-dependent antioxidant enzyme glutathione peroxidase 4 (GPx4), ferroptosis suppressor protein 1 (FSP1)-CoQ_10_, and dihydroorotate dehydrogenase (DHODH) have become a tripod complexion in ferroptosis regulation, which were summarized in **Figures 1**, **2**, and **Table 4**.

**Table 4:** The action of three main proteins regulating ferroptosis.

Proteins	Subcellular location about ferroptosis	Substrates	Function	Outcome	Reference
GPx4	Cytoplasm and cytosol, mitochondrion	Glutathione	An Antioxidant peroxidase lipid hydroperoxide	Preventing cells from ferroptosis	(119)
FSP1	Plasma membrane	NAD(P)H	Prevents lipid oxidative damage	Preventing cells from ferroptosis	(71, 120),
DHODH	Mitochondrion	Dihydroorotate	Catalyzes the conversion of dihydroorotate to orotate	Preventing cells from ferroptosis	(72)

GPx4, glutathione peroxidase 4; DHODH, dihydroorotate dehydrogenase; NAD(P)H, nicotinamide adenine dinucleotide phosphate; FSP1, ferroptosis suppressor protein 1.

GPx4, a GSH-dependent enzyme, is located in the cytoplasm and mitochondria of macrophages and reduces lipid hydroperoxides (L-OOH) to lipid alcohols (L-OH) by catalyzing GSH as shown in **Figures 2A, C**; therefore, GPx4 generally controls the iron-dependent production of lipid alkoxy radicals (L-O·) from L-OOH, which directly reduces lipid peroxidation that has been produced in the macrophage cell membrane. In our past work, macrophage iron and lipid retention aggravated AS *via* the autocrine formation of hepcidin in macrophages (121). Notably, the overexpression of GPx4 significantly lessens lipid modifications by the superoxide and impedes the progression of atherosclerotic plaque in ApoE^-/-^ mice (122), indicating that ferroptosis is a risk factor in the progress of CVDs. Breakthroughs have been made in cancer treatment, RSL3, ML162, ML210, 4-hydroxytamoxifen, and the FDA-approved anticancer agent altretamine could suppress GPx4 activity to induce cancer cell ferroptosis (120, 123). Although the evidence has shown that GPx4 participates in the formation of atherosclerotic plaques, the GPx4 knockout animal model, specific inhibitors, and activators of GPx4 may better unveil whether ferroptosis decisively participats in plaque formation in AS.

Recently, Doll et al. and Bersuker et al. simultaneously discovered that FSP1 is a potent protein resistant to ferroptosis independent of GPx4 (71, 120). The great discovery of FSP1 explains the dilemma in the tolerance of anti-cancer treatment by inhibiting GPx4 activation and provides new insights into the ferroptosis inhibition of macrophages in AS. FSP1 contains two mandatory domains for its function in suppressing ferroptosis, i.e., N-myristoylation and a flavoprotein oxidoreductase domain. FSP1 is recruited to the plasma membrane (endoplasmic reticulum, cytomembrane, and Golgi apparatus) by the key site N-myristoylation. Once recruited, FSP1 serves as an NAD(P)H-dependent oxidoreductase to reduce coenzyme Q_10_ (shown in **Figure 2B**). Coenzyme Q_10_ is an active lipophilic electron carrier that is the only one lipid-soluble antioxidant synthesized endogenously (124). Coenzyme Q_10_ plays a vital role in aerobic respiration to transfer electrons in mitochondria and keeps the lipids of Golgi and cell membranes away from oxidation (125). Although CoQ_10_ has been reported for decades in mitochondria, it is a novel discovery that CoQ_10_ is reduced by FSP1 at the cell membrane, which is sufficient to suppress the production of lipid peroxides and ferroptosis. Due to the special pathology of AS, local iron overload and lipid overload provide the necessary conditions for ferroptosis, and the FSP1-CoQ_10_-NAD(P)H pathway may be a promising strategy for inhibiting atherosclerotic plaque formation.

DHODH is a newly discovered protein operating parallel to GPx4 to inhibit ferroptosis in 2021. In the past decades, DHODH was regarded as an enzyme to catalyze *de novo* pyrimidine synthesis and produce uridine monophosphate, which would generate pyrimidines to biosynthesize nucleic acids for cell proliferation (125). In metazoans, DHODH is recruited to the outer face of the mitochondrial inner membrane, an environment rich in lipids, indicating that DHODH is a potential enzyme to inhibit ferroptosis by reducing lipid peroxidation (see **Figure 2D**). Because of the favorable permeability of the mitochondrial outer membrane, DHODH inserted into the outer membrane has access to its substrate dihydroorotate and mitochondrial inner membrane-bound CoQ. Subsequently, the product orotate of DHODH could be utilized in the *de novo* pyrimidine synthesis pathway in the cytoplasm (126). Therein, DHODH catalyzes dihydroorotate to orotate with quinone as an electron acceptor, which is an essential step for inducing ferroptosis.

Dihydroorotate and orotate are the substrate and product of DHODH. Mao and his colleagues reported that a separate supplementation with them could attenuate or enhance ferroptosis triggered by the inhibitors of GPx4 (72). When the GPx4 expression is inhibited, DHODH inactivation could induce substantial mitochondrial lipid peroxidation and trigger ferroptosis while cooperating with inducers to initiate ferroptosis in cancer cells with a high expression of GPx4 (72). DHODH inhibited ferroptosis independently of mitochondrial GPx4, cytosolic GPx4, and cytomembrane FSP1 in the mitochondrial inner membrane by reducing ubiquinone to ubiquinol, a radical-trapping antioxidant with anti-ferroptotic activity (see **Figure 2D**). Meanwhile, brequinar, the inhibitor of DHODH, selectively inhibited cell growth with low-expressed GPx4 by inducing ferroptosis. Furthermore, the authors reported that a combined treatment with brequinar and sulfasalazine synergistically triggered ferroptosis and suppressed tumor growth with a high expression of GPx4 as well (72). Unlike anticancer treatment, iron overload and lipid peroxidation are two key points for macrophage ferroptosis that should be avoided in AS, suggesting that except for the GPx4, FSP1 pathway, DHODH is a promising target for the suppression of macrophage ferroptosis in AS.

## Macrophage Autophagy Is a “Defender” in AS

Autophagy mediates the degradation and recycling of the damaged organelles and proteins *via* autophagy-related genes in lysosomes, which is a conserved process for maintaining cellular homeostasis. However, the dysregulation of autophagy has been associated with various metabolic disorders including AS. It was reported that autophagy could be triggered in macrophages, VSMCs, and endothelial cells (127).

In macrophages, autophagy could be induced by oxidized lipids, like ox-LDL and 7-ketocholesterol (128). The activation of autophagy could protect macrophages by digesting the damaged proteins, organelles, or lipids. Notably, lipophagy is selective autophagy that targets lipid droplets to lysosomes for degradation in macrophage foam cells (129). Defective autophagy in macrophages would promote the apoptosis and necrosis of macrophages, causing plaque instability in AS (80). Autophagy is generally regulated by LC3, autophagy-related genes (Atg), p62, AMPK, etc., and it could be regulated by its inhibitors and agonists in CVD, which were summarized in **Table 3**.

## Macrophages Pyroptosis and Instability Plaques

Pyroptosis, a programmed cell death, is closely associated with the activation of NLRP3 inflammasomes and the rapid release of various cytokines, such as IL-1β, IL-18, and HMGB-1 (130). In 2011, Kayagaki and colleagues reported that caspase-11 induces caspase-1-independent pyroptosis in macrophages, which is a noncanonical inflammasome pathway (131). Macrophage pyroptosis induced by the cholesterol crystal or ox-LDL promotes plaque destabilization. Significantly, NLRP3 inflammasome components are highly expressed in macrophages (132); therefore, inhibiting macrophage pyroptosis and reducing inflammation would provide prospective therapeutic strategies for the disease.

The NLRP3 inflammasome consisted of three parts: NLR (NOD-like receptor) families and PYHIN (pyrin and HIN domain-containing protein) families, ASC (apoptosis-associated speck-like protein), and the effector caspase-1. As shown in **Table 3**, the cholesterol crystal or ox-LDL is phagocytosed by macrophages, which facilitate NLRP3 inflammasome assembly and then activate caspase-1. Subsequently, activated caspase-1 cleaves pro-IL-1β to mature IL-1β, which induces inflammation and competes with cholesterol for access to the ABCA1, causing the retention of cholesterol to form foam cells (97). In addition, gasdermin D (GSDMD) could be cleaved by caspase-1 to N-terminal fragments (GSDMD-NT) and C-terminal fragments (GSDMD-CT) (133). GSDMD-NT translocates to the cytomembrane, self-oligomerizes, and forms the pores to disrupt the homeostasis of intracellular and extracellular osmotic pressure, leading to macrophage pyroptosis to form instability plaques (134).

## Conclusion and Prospect

In the past decades, the hypotheses and mechanisms of AS pathogenesis have always refreshed our perception. The knowledge of innovation on how the stabilization mechanisms that govern cholesterol and lipid transport and stay inside macrophages are operated to be foam cells helps us to tease out the initiating causes and has identified several pathological mechanisms, including inflammation, macrophage polarization, and macrophage death, which can regulate the formation of atherosclerotic plaques. According to the three classical hypotheses above, inflammation, lipid oxidation, and macrophage foam cells display major roles in AS progression, which reminds us to be concerned about the primary cause. Based on the past studies on AS, we summarized two main causes inducing inflammation, promoting foam cell formation and the formation and rupture of plaques, i.e., macrophage polarization and macrophage death.

As the disease progresses, plaques and the necrotic core are built up, although macrophage death is going on, most intense in advanced plaques yet. The recognition of disparate macrophage phenotypes (e.g., M1, M2, and Mox macrophages) has raised the question of who is responsible for foam cell formation the most because of the different abilities for phagocytosis, antioxidation, and death. Notably, it was reported that M1 macrophages could be resistant to ferroptosis compared to the M2 subtype (135). Furthermore, Mox may be an excellent anti-atherosclerotic subtype due to its abilities of weak phagocytosis and strong oxidation resistance. Macrophage death should be prevented, or dead cells should be removed effectively. From the clinical point of view, many therapies, such as statins, whose primary function is lowering serum LDL levels, are not particularly effective (136). It provides new insights that induce macrophages to polarize to anti-atherosclerotic subtypes as well as suppress macrophages’ death by the key molecules of apoptosis, necrosis, ferroptosis, autophagy, and pyroptosis. Taken together, macrophage polarization and death are two main archcriminals that are promising candidates for AS prevention and treatment.

## Data Availability Statement

The raw data supporting the conclusions of this article will be made available by the authors, without undue reservation.

## Author Contributions

All authors contributed to the article and approved the submitted version.

## Funding

This work was supported in part by the National Natural Science Foundation of China (No. 81973044, 82003448).

## Conflict of Interest

The authors declare that the research was conducted in the absence of any commercial or financial relationships that could be construed as a potential conflict of interest.

## Publisher’s Note

All claims expressed in this article are solely those of the authors and do not necessarily represent those of their affiliated organizations, or those of the publisher, the editors and the reviewers. Any product that may be evaluated in this article, or claim that may be made by its manufacturer, is not guaranteed or endorsed by the publisher.

## Abbreviations

AS, atherosclerosis; CXCL4, CXC chemokine ligand 4; CVD, cardiovascular disease; DHODH, dihydroorotate dehydrogenase; FSP1, ferroptosis suppressor protein 1; GPx4, glutathione peroxidase 4; LDL, low-density lipoprotein; L-OOH, lipid hydroperoxides; L-OH, lipids alcohols; MMP-7, matrix metalloprotease 7; Nrf2, nuclear factor E2-related factor 2; TrxR1, thioredoxin reductase 1; Srxn1, sulfiredoxin-1; VSMAs, vascular smooth muscle cells.
